# Supplementary figures and images for: Cystatin M/E Ameliorates Multiple Myeloma-Induced Hyper Osteolytic Bone Resorption
Source: Cancers (Basel). 2025 Feb 27;17(5):833. doi: 10.3390/cancers17050833 (PMC11899016; doi:10.3390/cancers17050833)

## Slide 1
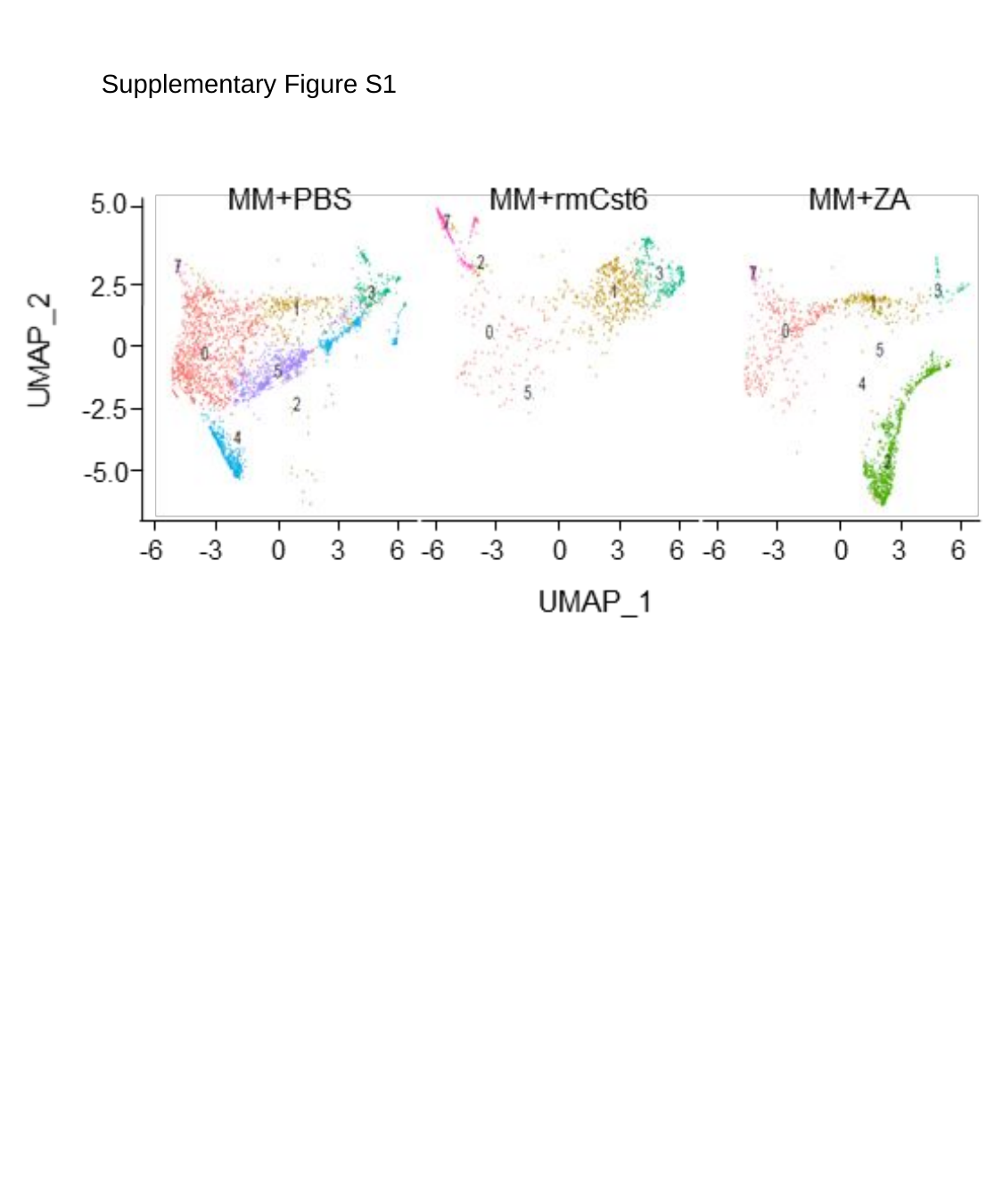

Supplementary Figure S1

Supplement: Supplementary file 1 [file cancers-17-00833-s001.zip › Supplemental figure S1 for CST6 ZA paper.pptx]
